# Thermally Activated Deformation Behavior of ufg-Au: Environmental Issues During Long-Term and High-Temperature Nanoindentation Testing

**DOI:** 10.1007/s11837-015-1638-7

**Published:** 2015-09-23

**Authors:** Verena Maier, Alexander Leitner, Reinhard Pippan, Daniel Kiener

**Affiliations:** Erich-Schmid-Institute for Materials Science, Austrian Academy of Sciences, Jahnstr. 12, 8700 Leoben, Austria; Department Materials Physics, Montanuniversität Leoben, Jahnstr. 12, 8700 Leoben, Austria; Materials Center Leoben, Rosenegger Str. 12, 8700 Leoben, Austria

## Abstract

For testing time-dependent material properties by nanoindentation, in particular for long-term creep or relaxation experiments, thermal drift influences on the displacement signal are of prime concern. To address this at room and elevated temperatures, we tested fused quartz at various contact depths at room temperature and ultra-fine grained (ufg) Au at various temperatures. We found that the raw data for fused quartz are strongly affected by thermal drift, but corrected by use of dynamic stiffness measurements all the datasets collapse. The situation for the ufg Au shows again that the data are only useful with drift correction, but with this applied it turns out that there is a significant change of elastic and plastic properties when exceeding 200°C, which is also reflected by an increasing strain rate sensitivity.

## Introduction

Due to their outstanding sensitivity regarding localized mechanical data, nanoindentation experiments have become quite popular in recent years and have been very successful in bringing further insights into the framework of small-scale deformation mechanisms.^1^,^2^ By improving different indentation methods, and by progressing from quasi-static to rate-dependent testing protocols, reliable and reproducible data on time-dependent processes can nowadays be acquired for different materials and microstructures; for example, enabling one to address thermally activated processes on a local scale.^3^ Thus, it has become possible to study the origin of rate-dependent properties on a microstructure-based level. However, the desired extension to harsh environments in terms of temperature,^4^–^7^ atmosphere,^8^ or other media,^9^ is still at the beginning of their development. This requires careful experimental design and detailed knowledge about the indentation system itself, as subtle changes in the testing system over time or temperature will affect the determined properties. Of particular importance is the notion that drift rates are not constant over long-term experiments,^3^ a common assumption in drift correction schemes that should be critically considered in long-term or high-temperature (HT) experiments. Besides the different instrument designs, described recently by Wheeler et al.,^10^ the selection of the correct indenter tip and indenter material is essential. In several studies, it has been shown that, with increasing the testing temperature, interactions between sample and tip take place, which can result in dissolution of the tip material, or oxide or carbide formation, thus degrading the initially well-defined tip shape.^11^ Moreover, the environmental conditions are of great importance, since surface oxides might significantly influence the experimental results.^12^ A great advantage is therefore the combination of HT testing and a vacuum environment, as published by Korte et al.^6^ and Wheeler et al.,^7^ in order to minimize oxidation and also thermal drift. Another way to minimize thermal drift influences would be the use of a reference tip on the sample surface.^13^ This, however, requires uniformly flat samples, which poses some limitations to general indentation experiments.

With regard to materials properties, the time-dependent deformation behavior of highly deformed and thus ultrafine-grained (ufg) materials has been of great interest within the last decade, since these materials can combine strength and toughness,^14^ but also exhibit enhanced rate-dependent properties.^15^ Applying different severe plastic deformation processes such as equal channel angular pressing (ECAP), accumulative roll bonding (ARB) or high pressure torsion (HPT), a fine-grained microstructure corresponding to the applied deformation strain can be reached. However, there is up to now a lack of knowledge and understanding of the dominating deformation processes.^15^–^19^ For face-centered cubic materials, it has been reported that the strain rate sensitivity, which describes the change in hardness/stress in response to the applied strain rate, is intensified for fine-grained structures.^15^,^20^ This behavior has been discussed in close relationship with thermally activated dislocation annihilation processes at high-angle grain boundaries,^15^,^21^,^22^ but also sometimes in context with grain boundary sliding, especially during localized hardness and nanoindentation testing. Overall, these studies are mostly performed at room temperature, and only in a very limited number of cases at elevated temperatures.^23^–^25^ Thus, a final discussion about the dominating factors is hard to conclude at the present state.

In this paper, we will focus on both the possible influences of experimental thermal drift issues on mechanical properties, at room and elevated temperatures, and also on the local deformation behavior, in terms of strain rate sensitivity, of the noble metal gold in an ufg condition also at room and elevated temperatures. Fused quartz (FQ), which is a standard calibration material, was chosen for a reference material, and several series of nanoindentation long-term creep/relaxation experiments were performed at room temperature for up to 2 h and also for varying indentation depths. These will be used to evaluate the possible influence of the applied indentation depth, thus the potentially changing heat flow through the contact area on thermal drift and thermal activation parameters determined by relaxation and creep kinds of indentation experiments. Moreover, elevated temperature nanoindentation experiments will be shown for ufg-Au. This material choice was guided by the fact that the rather soft noble metal eliminates concerns of sample oxidation, tip interaction and tip modifications by wear. Thus, we again aim to study only temperature-related influences on material properties, without complications from other potentially influencing factors. We show that, while standard material parameters such as hardness and Young’s modulus can be easily determined even at elevated temperatures, long-term experiments are very prone to be influenced by thermal changes, or contact heterogeneities, which will be discussed in detail.

## Experimental

Ufg-Au disks with a diameter of 8 mm and 0.8 mm in thickness were produced via a powder metallurgical HPT route,^12^,^26^,^27^ in which spherical Au powders with a diameter of ~7 *µ*m and purity of 99.96% were compacted by HPT anvils and subsequently subjected to the severe plastic shear deformation.

Cross-sections of the ufg-Au disks were prepared for metallographic examinations and nanoindentation testing. The specimens were mechanically ground, subsequently polished with diamond suspension down to 1 *µ*m, and finally electrolytically polished in order to remove any potentially remaining deformation layer. Subsequently, the grain structure was analyzed using the electron backscatter diffraction (EBSD) technique in a scanning electron microscope (Leo1525; Zeiss, Oberkochen, Germany). The median grain size of the ufg material before and after HT testing was evaluated using the automated grain determination routine of the EBSD software OIM TM data analysis (EDAX, Mahwah, USA). A misorientation angle of 15° was set to differentiate small-angle from high-angle boundaries at a step size of 15 nm.

Nanoindentation experiments were conducted using a Nanoindenter G200 (Keysight Technologies, USA) equipped with a continuous stiffness measurement (CSM) unit and a three-sided Berkovich pyramid [material selection: for room temperature (RT), diamond (MicroStar, USA); for elevated temperatures (HT), Sapphire (Synton, Switzerland)]. Machine stiffnesses and tip shape calibrations were performed at room temperature according to the Oliver–Pharr method,^28^ and nanoindentation testing was carried out at room temperature (28°C; room not air-conditioned) as well as at high temperatures of up to 400°C. Elevated testing temperatures were realized using a Laser Heating Stage (SurfaceTec, Hückelhoven, Germany), in which tip and sample are independently laser-heated to adjust and stabilize the contact temperature, minimize thermal drift influences, and guarantee a well-defined homogenous temperature distribution during the entire indentation process. Moreover, during the HT experiments, the whole system is actively water-cooled to 18°C, including the HT sample tray as well as a Cu cooling shield which is arranged around the heated tip in order to protect the indenter head from thermal influences. In addition, an inert gas environment (forming gas: N_2_ containing 5% H_2_) was created by two separately controlled valves that allow the individual regulation of the gas flow at the sample tray and in the cooling-shield, thereby creating an oven-like, gas flooded volume around the tested sample. Obviously, for materials prone to oxidation, this would also prevent oxide formation. Finally, the initial thermal drift for all testing temperatures was set to <0.3 nm/s before starting the experiments, and the actual drift rates were determined previously for each indentation array, with typical values in the range of 0.1 nm/s or better for all temperatures.

In terms of indentation protocols, standard constant strain rate (cSR-CSM) experiments (0.025 s^−1^) were carried out up to 2500 nm indentation depth. Furthermore, the local strain rate sensitivity (SRS) was measured using nanoindentation strain-rate jump tests,^3^,^25^,^29^ applying reversible strain rate changes from 0.025 s^−1^ to 0.005 s^−1^, or 0.0025 s^−1^. The rate changes were performed at 500 nm and 1500 nm, respectively, and back to the initial strain rate at 1000 nm and 2000 nm indentation depth. The chosen strain rates are equivalent to indentation strain rates of 0.05 s^−1^, 0.01 s^−1^ and 0.005 s^−1^$\left( {{{\dot{h}} \mathord{\left/ {\vphantom {{\dot{h}s} h}} \right. \kern-0pt} h} = \frac{1}{2}{{\dot{P}} \mathord{\left/ {\vphantom {{\dot{P}} P}} \right. \kern-0pt} P}} \right)$, respectively.^30^ For all strain rate controlled tests, the CSM frequency was set to 45 Hz and a harmonic amplitude of 2 nm was superimposed. At room temperature, six, and at elevated temperatures, four, indentations were performed for each indentation method and temperature. For long-term indentation relaxation tests, a procedure detailed in^3^,^23^ was used, which is based on ideas described in.^31^,^32^ Therefore, a strain rate-controlled indentation loading segment was performed to a preset relaxation start displacement, varying between 200 nm and 2000 nm for FQ, and at a fixed value of 1000 nm for ufg-Au. Afterwards, the apparent raw load was held constant for 2 h (RT experiments) and 30 min (elevated temperatures), while the CSM unit was continuously recording the contact stiffness during the entire holding segment with an amplitude of 2 nm and 45 Hz. Since the resultant, original displacement data are greatly influenced by varying thermal and experimental drift over time, the raw data were dynamically corrected for the actual drift rate using the measured contact stiffness and assuming a constant reduced modulus (determined during the indentation segment) (for further details of the correction method, the reader is referred to^3^,^23^). Since the original stiffness data showed some homogenous scatter, the corrected displacement and hardness values were smoothed by a three-parameter power-fit $\left( {y = a + b \cdot x^{c} } \right)$ using the SigmaPlot software. Resultant creep rates were calculated using $\dot{\varepsilon }_{\text{relaxation}} = {{\dot{h}} \mathord{\left/ {\vphantom {{\dot{h}} h}} \right. \kern-0pt} h},$ and the true hardness, based on the contact stiffness, was evaluated via $H = P/A_{{{\text{c}} - {\text{cor}}}}$. For very shallow indentation depths, the CSM amplitude could lead to a loss of contact between sample and tip.^33^ However, as our relaxation experiments started from indentation depths of at least 200 nm, we do not expect such influences.

To quantify the acting thermally activated processes, reflected by strain rate-dependent changes in hardness, the local SRS, *m*, can be determined as $m = \partial \ln H/\partial \ln \dot{\varepsilon }$. For strain rate jump tests, this leads to single data points as a function of material hardness, *H*, while for relaxation experiments continuous *m* values over *H* can be calculated.^3^,^23^

## Thermal Drift Issues During Room Temperature Nanoindentation: Strain Rate Sensitivity of Fused Silica

**Fig. 1:**
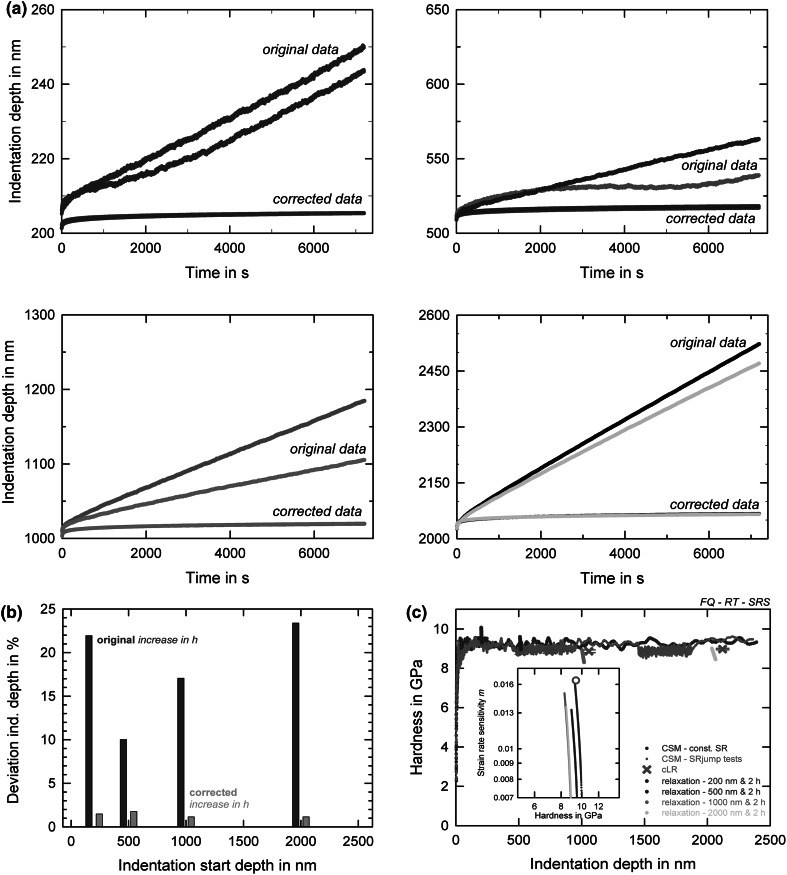
Environmental issues during long-term nanoindentation testing on fused silica at room temperature. (a) Raw data and drift-corrected creep displacement versus 2 h constant load time for different indentation depths (200 nm, 500 nm, 1000 nm, 2000 nm, from left to right). All *y*-axis represent an increase of 30% of the initial preset indentation depth. (b) Percentage of displacement increase for single indentations; original (red) and corrected (yellow) indentation data. (c) Hardness versus indentation depth for different indentation methodologies (cSR-CSM, strain rate jump,^3^ cLR as well as relaxation tests). The corresponding strain rate sensitivity is shown as inset

The measured indentation depth during long-term nanoindentation experiments is highly influenced by different factors. Figure 1a shows experiments on fused silica that lasted for 2 h each, where the load hold segment was started at different indentation depths of 200 nm, 500 nm, 1000 nm, and 2000 nm, and the subsequent changes in the displacement were recorded continuously. Obviously, for all different starting indentation depths, the original displacement data differ significantly, both for different creep starting depths and for different indents at the same condition, and do not show any general accordance to each other. For example, the maximum indentation depth between two tests starting at the same depth varies significantly, especially pronounced for the more shallow 200 nm, 500 nm, and 1000 nm creep experiments (note the different scaling on the *y*-axis of Fig. 1a). This indicates that there is no clear correlation between contact area and the extent of thermal drift influence. Moreover, the displacement data show a wavy, irregular behavior over the 2-h time period, confirming some environmental influences during displacement recording. However, the quantification of the thermal drift, which is determined at the end of each indent, only gives values between −0.003 nm/s and −0.061 nm/s for all different depths, showing thereby no clear relationship between any testing parameters. Note that uniform drift over 2 h would result in displacements in the range of 20–440 nm. Notably, these values again do not correlate with the raw displacements during the hold segment, proving that the final drift is not indicative for the whole experiment, although this is commonly assumed. However, correcting the displacement data by using the continuously measured contact stiffness as outlined before, for each different starting displacement, both curves are perfectly alike and lie on top of each other in Fig. 1a.

To quantify the increase of indentation depth during the hold period, and also the possible error that influences the data without correction, we note first that all shown diagrams represent a displacement range from the start of the creep segment to a 30% displacement increase. Due to the self-similarity of the pyramidal Berkovich tip, one would in fact expect that all data should be similar and independent of the initial indentation depth, as such material properties should be independent of the analyzed volume. For the original data, this is clearly not the case, strong fluctuations and varying trends are observed. However, after correcting for drift-attributed displacement, the creep response of the material under the self-similar tip is clearly the same, independent of initial creep depth, and applied load, respectively, as shown in Fig. 1a. The relative deviation of indentation depth after 2 h indentation relaxation is shown in Fig. 1b. Here, the percentage of indentation depth increase in respect to the start displacement is plotted over the indentation start depth. For the corrected data, the deviations are depth-independent as would be expected and show an increase between 1.15% and 1.77%. For the uncorrected, original data, these deviations are significantly more irregular, but again without any decisive, depth- or load-dependent trend. While the 200 nm and 2000 nm experiments exhibit increases of more than 20%, the 500 nm and 1000 nm tests range between 10% and 17%. For all indentations, the temperature difference recorded with a thermocouple inside the indentation chamber changes within less than 0.1°C during the 2 h experiment, showing only very slight variations. This, however, might already influence the displacement signal. As shown in previous works, such small differences influence the overall indentation displacement for long-term experiments,^3^,^23^ since the displacement for all nanoindentation systems is not measured directly at the sample, but using a sensor inside the indenter head, which for the used system is a capacitive kind. This results in a displacement signal that is a combination of pure material answer and thermal expansion of the indenter.

In Fig. 1c, results combining different nanoindentation methods on FQ are displayed. Conventional cSR indentations agree well with cLR indentations but also with the same strain rate segments of the strain rate jump tests. Additionally, the corrected hardness over indentation depth is plotted for the different relaxation experiments described in the previous paragraph. All data agree well with each other, indicating a depth independent hardness of 9 GPa and a very low SRS. We only noted a slightly increased hardness for small indentation depths in the relaxation experiments. The corresponding SRS is displayed in the inset of Fig. 1c, showing that fused silica exhibits only a slight SRS in a range of 0.007–0.016. This value is consistent with recent literature,^34^ showing that a careful experimental design and stable environmental conditions have to be guaranteed in order to get reliable and trustable results using long term nanoindentation, even at room temperature where thermal drift issues might be considered least influential.

## Deformation Behavior of ufg-Au: From Ambient to Non-ambient Conditions

Under non-ambient conditions, thus in harsh environments relevant to, for example, areas such as energy, mobility, transportation, tooling, to name just a few, influences of thermal drift are potentially strongly intensified. In all these fields, a general trend is to create stronger materials by reducing the grain size to the ultra-fine grained or nanocrystalline regime. Therefore, ufg-Au was investigated by nanoindentation testing techniques ranging from RT up to 400°C, thus up to 50% of the material melting point. As outlined before, we expected a pronounced influence of testing temperature on the deformation behavior of this ufg material, and, by choice of this noble metal, we can on purpose eliminate any other influencing factors emerging, for example, from oxidation or sample–tip interaction.

**Fig. 2:**
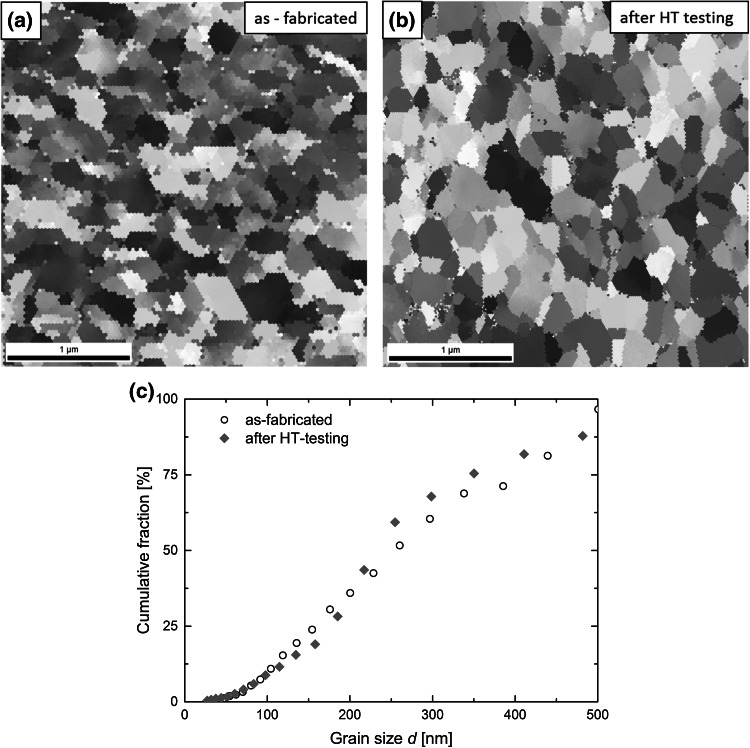
Microstructure of the ufg-Au as-received and after HT-testing. (a) Inverse pole figure map of the as-fabricated material and (b) after HT-indentation testing. (c) Results of the line intersection method: the median grain size is about the same for the initial as-fabricated state (291 nm ± 98 nm) and the HT-state (289 nm ± 101 nm)

In Fig. 2, the microstructure of the HPT-deformed ufg-Au is shown before and after finalizing nanoindentation testing experiments at 400°C, as well as the results from the line intercept method in order to quantify the microstructural changes. While the as-fabricated HPT material shows an initial grainsize of 291 nm ± 98 nm (Fig. 2a and c), after HT testing and more than 10 h at elevated temperatures, the grain size stayed about the same around 289 nm ± 101 nm (Fig. 2b and c), indicating that no significant grain coarsening took place.

**Fig. 3:**
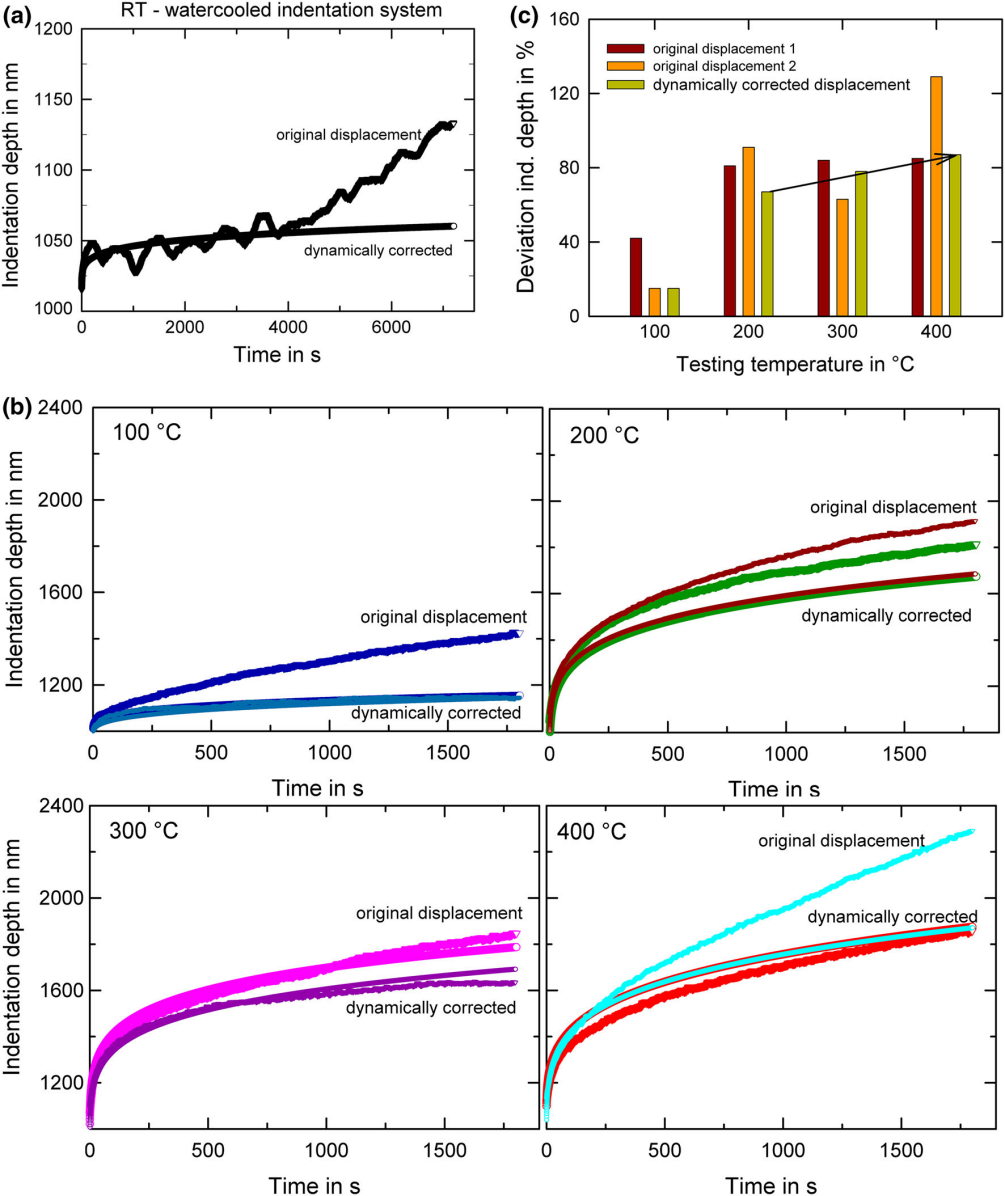
Nanoindentation relaxation tests on ufg-Au starting at 1000 nm indentation depth for constant load times of 2 h or 30 min. (a) RT, and (b) 100°C, 200°C, 300°C, and 400°C, showing original and dynamically corrected indentation data, respectively (with increasing creep influences with increasing T). (c) Percentage increase in indentation depth depending on temperature: original displacement of two individual indentations (left, red and middle, orange bars) and dynamically corrected (right, light green bar) displacement

Besides the influence of time and temperature on the original displacement data, temperature-related changes of hardness and Young’s modulus, as well as the determination of thermally activated processes, was part of the focus of this experimental part and will be discussed accordingly. The resultant raw displacements during nanoindentation relaxation experiments for 2 h at RT and 30 min at elevated temperatures, as well as the drift-corrected data and the percentage increase in displacement for the ufg-Au are shown in Fig. 3. At RT, the original signal is quite irregular, while the temperature inside the indenter chamber shows only a very gentle decrease from 23.03°C to 23.00°C (Fig. 3a). Since the overall environmental temperature is lowered by the cooling system by 5°C in comparison with the fused silica RT tests, this indicates that the water-cooling system significantly decreases the temperature inside the chamber. Moreover, this demonstrates that a small temperature change of the whole chamber is not the main origin of thermal drift, but, rather, local variations near the sample/tip contact due to convection might influence the original displacement signal more strongly. Increasing the testing temperature, the chamber temperature remained rather constant around 23°C due to the water cooling unit, while tip and sample were heated to different temperatures up to 400°C for several hours. Note the different range of creep displacements in Fig. 3 between RT and HT experiments. The original displacement signals are again influenced by thermal drift; however, the differences between the original and corrected data is less developed compared to the not actively cooled standard stage (Fig. 1). Some of the indentations shown even exhibit no significant difference between the original and corrected data; see, for example, some 100°C and 400°C experiments. This can also be seen in Fig. 3c, where the percentage of the increase in indentation depth is plotted against temperature for original and dynamically corrected data. Again, an overall reproducible trend cannot so far be seen. We relate this to two main aspects. Firstly, there are thermally more stable conditions inside the chamber due to the active cooling of stage and cooling-shield, leading to a reduced influence of other environment issues. Secondly, the more rate-dependent behavior of ufg materials leads to larger material intrinsic creep displacements, which can, for low enough drift contributions, dominate the recorded overall displacement. Notably, the original creep displacement of FQ at RT was 1050 nm (dynamically corrected 1015 nm) after 1800 s (Fig. 1: *h*_start_ = 1000 nm), while that of ufg Au was around 1050 nm for both datasets. It is easy to see that even a quite low constant drift rate of ~0.03 nm/s would lead to ~50 nm drift-related displacement during the experiment. While this is already a significant fraction compared to the creep-related displacement of the strain rate-dependent ufg Au, it is plainly clear that this hopelessly dominates over the 15 nm creep contribution of the FQ.

**Fig. 4:**
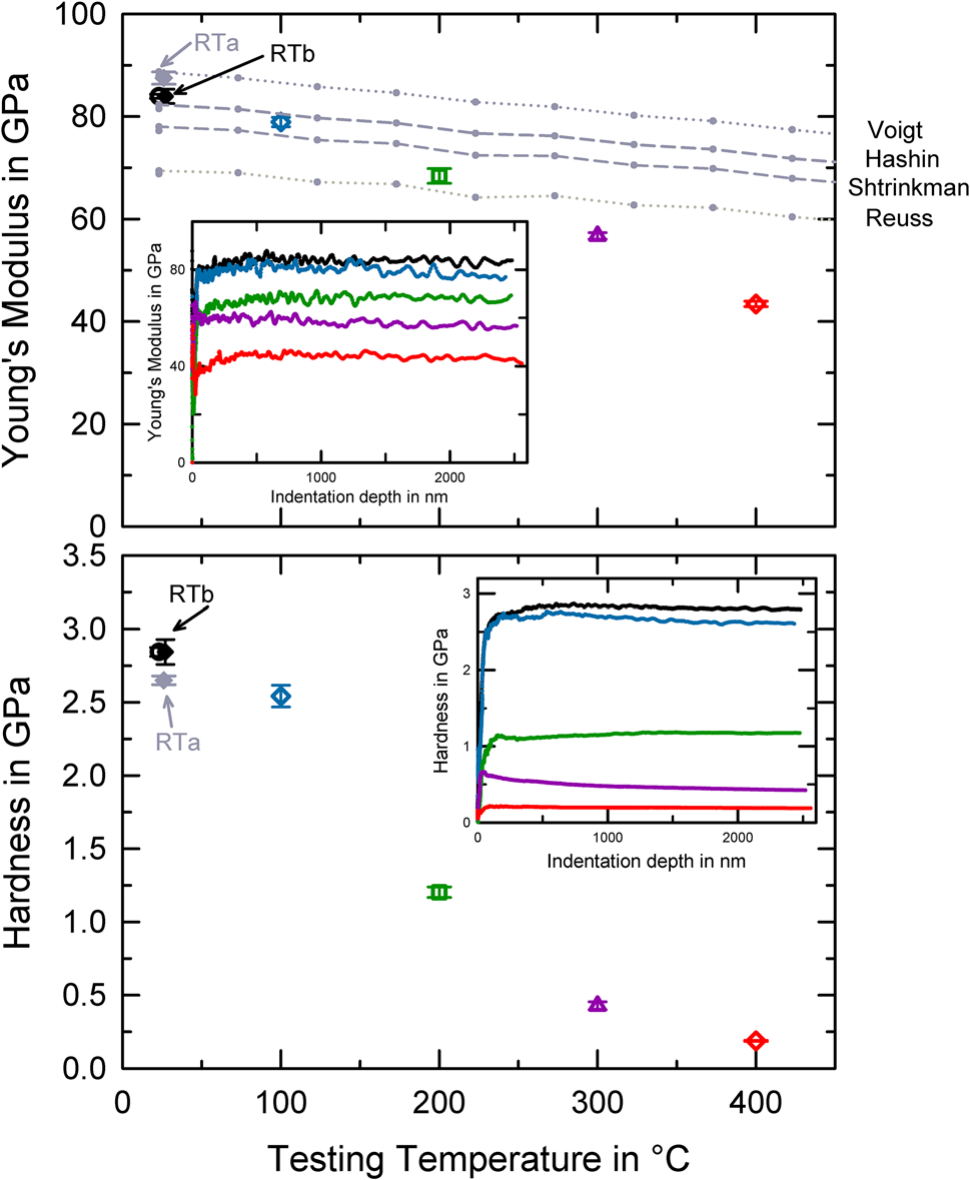
Temperature-dependent mechanical properties of ufg-Au. Hardness and Young’s modulus as a function of testing temperature, including literature data for sx-Au with upper and lower bounds of the Young’s modulus.^35^ The insets show depth-dependent hardness and modulus for each temperature separately, for RT test before (RTb: black circle for sapphire tip HT setup; black rhombus diamond tip) and RT tests after HT (RTa: gray rhombus indication for diamond tip) are displayed

The resultant mechanical properties determined at RT and elevated temperatures are shown in Fig. 4. The Young’s modulus and hardness investigated by cSR-CSM indentations (Fig. 4) both decrease with temperature, in a quite linear manner for the modulus. Thereby, the depth-dependent modulus for each indent remains constant over the indentation depth for all temperatures (see inset in Fig. 4), indicating that the machine stiffness is well calibrated and no stiffness loss in the contact was evident. Starting from 83 GPa at RT, the modulus, *E*, of the ufg-Au decreases with increasing *T* to 43 GPa. Cooling the sample to RT, the modulus increased back to 87 GPa. The data at RT and 100°C are clearly within the upper and lower bounds of the Young’s modulus published in literature.^35^ Overcoming 200°C, however, the modulus decreases more extensively than what would be expected from either the macroscopic polycrystalline or single crystalline literature data.^35^ Using HT nanoindentation, Beake et al.^4^ reported higher modulus data for gold at temperatures of 200°C (74.1 GPa) and 400°C (68.8 GPa) compared to the present results on ufg-Au. On the other hand, using indentation experiments at RT, Kiely and Houston^36^ found strong anisotropy effects in the modulus of Au, leading to differences between 85 GPa (111), 82 GPa (110), and 57 GPa (001).

There are different possibilities that can be considered to explain this unexpected behavior. Firstly, in terms of a real material effect in the ufg-Au, the modulus might decrease more extensively with *T* than for coarse-grained or single crystal materials, because the high amount of grain boundaries in the plastic zone leads to a higher compliance of the material. Generally, this was found for materials with grain sizes in the nanocrystalline regime, but has not yet, to our knowledge, been conceived for ufg materials, as the amount of grain boundary volume appears to be a quite small fraction of the total material volume for ufg grains to expect noticeable effects.^37^–^39^ A possible scenario explaining this modulus reduction above 200°C might be an increasing grain boundary volume with increasing temperature. This is conceivable when considering that, at these homologous temperatures exceeding 0.35 (for Au at 200°C), climb-related accommodation processes of dislocations at grain boundaries occur ever faster, leading to an increased vacancy concentration in the grain boundaries.

Secondly, in terms of experimental reasons, some machine compliance or sample fixation problems may influence and affect the results. However, since the modulus data shown in Fig. 4a is constant over indentation depth for all temperatures, this seems somewhat unreasonable, as a constant modulus over temperature is generally a good indicator for a stable and reliable sample fixation. Additionally, we also tested a flat sample placed just under the indenter without fixation. In that case, as expected, a completely unreproducible and changing modulus resulted. Furthermore, we took efforts to confirm the actual residual impressions of representative indents. One indent for each temperature condition and testing method were imaged in an SEM. Subsequently, the projected area was measured using the free software ImageJ and compared to the values obtained by the nanoindenter software, NanoSuite. The deviations are represented in Table I.

**Table I:** Deviations of projected areas between software values and values determined by measurements of residual impressions with an SEM for the testing methods and temperatures used

	RT (%)	100°C (%)	200°C (%)	300°C (%)	400°C (%)
CSM	6	16	−6	0	−8
JUMP	12	14	13	10	−9
CREEP	15	7	−4	12	−74

This examination results in an unsystematic error with an average of 10% deviation when the 400°C indent is not considered (see strong required correction due to higher drift in Fig. 3). However, it must be noted that the SEM measurement of the indents is still prone to errors due to the limited possibility to determine the exact position of the rounded corners of the impressions.

However, an unassailable proof regarding experimental influences cannot be made at this point and further investigations to support our working hypothesis and to eliminate experimental concerns are on their way.

The measured material hardness as a function of testing temperature is shown in the lower part of Fig. 4. With increasing temperature, the hardness decreases from 2.8 GPa at RT before testing to 0.2 GPa at 400°C. Nanoindentation testing at RT after the HT environments delivered values of 2.65 GP, slightly lower than that found for testing of the as-received material. These results fit perfectly to the microstructural evolution taking place, showing slight coarsening effects (Fig. 2). The HT data are again surprisingly low considering the slight changes observed in microstructure (Fig. 2), and also in comparison to the literature data.^4^ However, there is not an extensive body of work, as only a very few studies including HT properties of ultrafine-grained Au exist. Beake et al.^4^ investigated Au and reported hardness values of 0.38 GPa at 400°C, but the chemical composition and microstructure are not stated in this reference, which hinders a reasonable comparison. On the other hand, it is known that the hardness decrease in ufg materials can be more pronounced than in single crystal or coarse-grained materials, which is attributed to the increased SRS. This was, for example, investigated for ufg-Al and cg-Al by May et al.^20^ who reported that, for low strain rates of around 10^−6^/s, the ufg-Al deformed at even lower stresses than the cg-Al reference material. Notably, for higher strain rates, this was not the case in Al.

Nonetheless, considering the constant hardness over indentation depth as shown in the lower inset of Fig. 4, and more importantly the constant modulus indicating a good quality experiment, we think that this pronounced softening at elevated temperatures has to be a material effect. As mentioned above, taking into account the large amount of grain boundaries present in the plastic zone, it is not too surprising that at elevated temperatures an increasing amount of accommodation processes takes place, thereby removing possible dislocation pile-ups and related back-stresses on dislocation sources, which effectively reduces the material hardness.

**Fig. 5:**
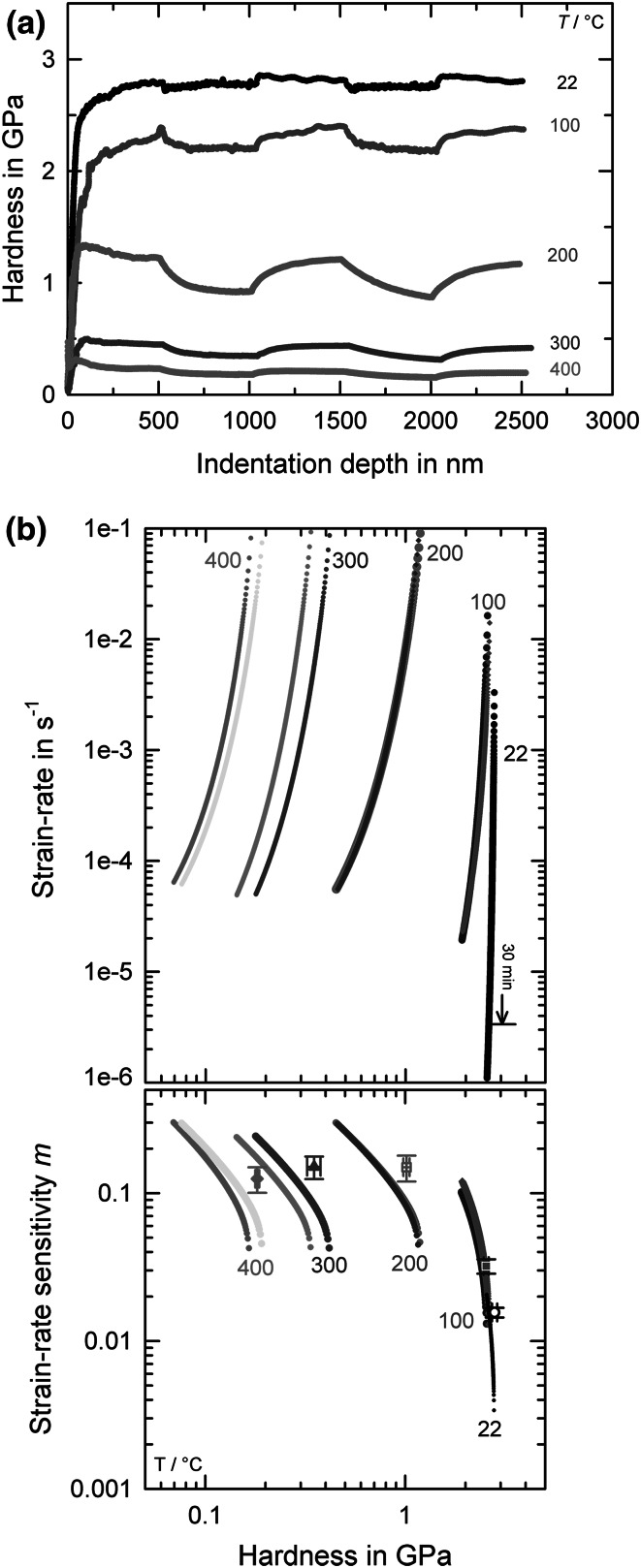
Thermally activated processes in ufg-Au. (a) Nanoindentation strain rate jump tests for varying temperatures. (b) Norton plots determined from nanoindentation relaxation tests (30 min holding time for HT experiments) and the corresponding time- and temperature-dependent strain rate sensitivity. Additionally, results from strain rate jump tests are included

Focusing on the strain rate sensitive deformation behavior, nanoindentation strain rate jump tests were also performed for all temperatures (Fig. 5a). Obviously, the hardness values decrease similarly to that determined in cSR-CSM indentations, but the applied strain rate changes lead to distinct jumps in the hardness along the indentation curve. With increasing temperature, these jumps not only become intensified but also the curvature describing the transient behavior changes significantly, starting with distinct jumps exhibiting negligible transient behavior at low temperatures to extensive curvatures at elevated temperatures. This behavior is in close agreement with experiments performed on ufg-Al.^24^ The resultant SRS *m* is plotted in Fig. 5b combined with the dynamically corrected relaxation results (Fig. 3). The Norton plots, where the calculated creep/relaxation rates are shown as a function of the material hardness, also depict the extensive hardness decrease. With increasing temperature, the curvature becomes more pronounced, showing a more extensive hardness decrease paired with higher relaxation rates. For each testing temperature, two different indents are shown which are mostly in good accordance to each other (only *T* = 300°C shows a small discrepancy). The corresponding SRS, *m*, which is evaluated from the slope of the Norton plot, is shown in the lower part of Fig. 5b. It is seen that *m* increases with increasing testing time as well as increasing testing temperature. Moreover, the slope of the creep curve is intensified at higher *T*, leading to higher *m* values. Additionally, the SRS determined using strain rate jump tests (Fig. 5a) is also plotted. For all temperatures, it appears that the hardness values are in good accordance between the two testing methodologies; however, for higher *T*, relaxation tests lead to slightly lower SRS values at similar hardness levels. This could be due to some difference in microstructure, but is more probably due to different applied strain rates during both testing techniques.

What is interesting to note with respect to the temperature-dependent deformation behavior of the ufg-Au is that, at temperatures ≥ 200°C, the SRS is substantially increased by an order of magnitude compared to the RT value, almost reaching values found for superplastic deformation behavior.^40^ This again indicates a pronounced increase in thermally activated accommodation processes of dislocations at grain boundaries. Moreover, this also corresponds to the temperature at which we observed an increased reduction of the elastic modulus, which would support our working hypothesis regarding the modulus reduction at elevated temperatures by increased grain boundary volume.

From a broader perspective, the rate-dependent behavior is in good agreement with other reports on fcc ufg materials in the literature. Reference data for pure ufg-Au itself are very rare,^41^,^42^ since only a limited number of studies have been performed to date. Merle et al.^41^ recently found significantly higher *m* values for nc Au films investigated via small-scale bulge testing. However, there were some differences in the materials’ purity, since the tested material was highly pure and additionally in the form of a ~300-nm-thick free-standing film in nature. Investigating an Au film supported by a SiN_*x*_ substrate, the SRS was found to be in the same range as in this study. Moreover our material is, owing to the powder production route, not as pure as the thermally evaporated Au coated on SiN_*x*_. However, Nyakiti et al.^42^ investigated different Au-Cu alloys and found for Au with only 1 wt.% Cu similar SRS as in our tests. This seems reasonable, since via our chosen production route, small amounts of Cu are incorporated as impurities. In order to allow a further comparison with other fcc materials, ufg-Cu seems to be a reasonable choice, since the melting point and thus the homologous temperature of both are almost the same. Generally, for ufg-Cu, a quantitatively similar behavior has been reported,^21^ relating the increased SRS with thermal activation of dislocation motion and their annihilation at high-angle grain boundaries.

## Critical Remarks and Outlook: Influences During Elevated Temperature Long-Term Nanoindentation

Generally, we want to emphasize that a profound knowledge about the environmental conditions, but also a reliable and robust control of the stability of the testing system itself over time and temperature, is crucial. As shown in this work, but also already stated in the literature several times before, small changes in the local temperature might lead to significant errors in the displacement signal of the system. This obviously becomes more pronounced the longer the measurement takes, thereby natively affecting relaxation kinds of experiments more severely. While the choice of materials used here was deliberately to avoid environmental effects, influencing environmental conditions such as oxidation or tip degradation must be kept in mind in order to allow a useful and meaningful discussion of nanoindentation results under non-ambient conditions. Although we deliberately avoided any other factors, it turns out that just the influence of local thermal drift itself results in completely irregular material response with no correlation between creep displacement, creep depth, or contact area. This time-dependent variation of local drift rates can, in our opinion, be efficiently dealt with by using a continuously recorded contact stiffness, such as the CSM or CMX techniques, to correct for the true contact area. Vice versa, long-term data without this kind of correction possibility should be considered critically. For future developments and a deeper understanding of possible machine effects, it would be interesting to compare well-characterized reference materials such as the ones used in this work on different platforms using a defined set of testing protocols.

Turning to the HT experiments, the drift influences remain the same. The only difference is that the SRS typically increases with temperature, which means from a practical point of view that larger creep displacements will occur, while the drift contribution might remain similar, which gives a better ratio between material contribution versus drift influence to the total displacement measured. There appears to still be some difference between jump tests and creep tests, but this might relate to the different stresses and strain rates where the SRS was derived. This can be improved by experimental design and data analysis.

Aiming for ever increasing temperatures, it is clear that oxidation and tip interaction will be the main challenges, and possibly the most promising direction is moving towards vacuum conditions. Still, remaining drift influences will occur and will require correction. Thus, as CSM seems to be a potent method for doing so, it might be worth looking carefully for possible influences of the CSM data versus static nanoindentation data for all indentation depths.
